# Lysolecithin-derived feed additive improves feedlot performance, carcass characteristics, and muscle fatty acid profile of *Bos indicus*-influenced cattle fed in a tropical environment

**DOI:** 10.3389/fvets.2023.1041479

**Published:** 2023-03-30

**Authors:** Rodrigo Dias Lauritano Pacheco, Jessica Oliveira Gusmão, Gustavo André Moura, Matheus Capelari, Leandro Greco, João Carlos Fontanari de Carvalho, Rafael da Costa Cervieri, Patrick André Castro, Valquíria de Alencar Beserra, Victor Paschoal Consentino Campanelli, Luciano da Silva Cabral, Laura Barbosa Carvalho, Dante Pazzanese Duarte Lanna, Marcos Chiquitelli Neto, Michael Galyean, Alex Sandro Campos Maia

**Affiliations:** ^1^Agro-Pastoril Paschoal Campanelli, Altair, SP, Brazil; ^2^Laboratory of Bioclimatology, INOBIO-MANERA, Department of Animal Science, São Paulo State University, Jaboticabal, São Paulo, Brazil; ^3^Kemin Industries, Valinhos, São Paulo, Brazil; ^4^Nutribeef Consulting, Botucatu, São Paulo, Brazil; ^5^Department of Animal Science, Federal University of Mato Grosso, Cuiabá, Mato Grosso, Brazil; ^6^Laboratory of Animal Nutrition and Growth, Department of Animal Science, University of São Paulo, Piracicaba, São Paulo, Brazil; ^7^Department of Biology and Animal Science, São Paulo State University, Ilha Solteira, São Paulo, Brazil; ^8^Department of Veterinary Sciences, Texas Tech University, Lubbock, TX, United States

**Keywords:** feed additives, lysolecithin, zebu, phase-feeding, Nellore, heat stress

## Abstract

Lysolecithin might increase ruminal and intestinal emulsification, leading to increased digestibility, but there is minimum information about which is the most appropriate phase to start supplementation and its impacts on feedlot performance and muscle fatty acid profile. Two experiments were conducted to evaluate the effects of phase-feeding of Lysoforte™ eXtend (LYSO). In the first experiment, 1,760 predominantly *Bos indicus* bullocks (initial body weight of 400 ± 0.561 kg) were allocated in a complete randomized block design. LYSO was supplemented at 1 g/1% of ether extract from the diet. Treatments were no LYSO supplementation (NON); LYSO starting during the growing period and continuing during the finishing period; LYSO starting during the finishing period (FIN); and LYSO during adaptation, growing, and finishing periods (ALL). In the second experiment, the same treatments were evaluated with 96 bullocks (64 Nellore and 32½ Nellore × ½ Angus) in a 4 × 2 factorial arrangement (treatments × genotype). For both studies, intake and average daily gain were accessed; carcass characteristics were evaluated in the first experiment, while digestibility of nutrients and profile of muscle fatty acids were measured in the second experiment. In the first experiment, LYSO increased final body weight (*P* < 0.022) and average daily gain (GRO and FIN; *P* < 0.05). In the second study, a treatment × breed × feeding phase interaction was observed with Nellore having a greater average daily gain (*P* < 0.05) than crossbreds in every feeding phase that LYSO was introduced to the diet. A treatment × feeding phase interaction was observed for digestibility, such that LYSO increased total dry matter (*P* = 0.004), crude protein (*P* = 0.043), and NDF (*P* = 0.001) digestibility during the finishing period. A treatment × breed × day classification was observed (*P* < 0.05). During the finishing phase, crossbreds treated with LYSO had greater DMI (*P* < 0.05) on very hot days than NON. Also, animals treated with LYSO presented a greater C18:3 n3 concentration (*P* = 0.047) in the *longissimus*. Overall, feeding LYSO during GRO and FIN enhanced feedlot performance and should lead to higher intakes during very hot days of the finishing feeding period.

## 1. Introduction

Fat inclusion is a worldwide nutritional strategy recommended by nutritionists (1–3), especially as a means of increasing dietary energy density. Moreover, a fraction of rapidly fermented carbohydrates can be replaced when fat is added to finishing diets, thereby decreasing the risk of metabolic disorders (4, 5).

Generally, fat sources can be classified as non-protected (tallow, yellow grease, vegetable oils, and seeds such as whole cottonseed) or protected (rumen bypass sources, which are mainly calcium salts of soybean and palm oil). Limitations to fat digestion and absorption exist and are inherent in the ruminant animal (6). In the rumen, microorganisms perform biohydrogenation of unsaturated fatty acids into more saturated forms (7), whereas bypass sources are emulsified and digested in the intestines. Independent of the site, microbial and host animal metabolism and digestion of fatty acids have limits that decrease the potential for fat inclusion in diets (8). Therefore, if lipid digestive capacity can be improved in ruminants, feed conversion, and costs could be decreased in feedlot operations.

Lysoforte^TM^ eXtend (LYSO) is an emulsifier additive composed of lysolecithin, synthetic emulsifier, and monoglycerides that increase lipid emulsification and emulsion stability (9), which can lead to higher fat digestibility, alter ruminal biohydrogenation, and increase ruminal lipid passage rate associated with the liquid phase (10). Hence, it may also lead to muscle fatty acid metabolism alterations in feedlot animals, as a greater flux of dietary unsaturated fatty acids is expected to be available for absorption in the intestines. Rumen-protected fatty acids sources, such as calcium salts, have been previously reported to increase feedlot performance (11), downregulate mRNA expression of enzymes related to lipid metabolism (12), and improve marbling (13). However, it has not yet been described how emulsifiers alter carcass characteristics, especially the *longissimus* fatty acids profile.

Calves receiving LYSO had improved fat digestion and absorption of nutrients (14). Also, in lactating cows, greater milk fat yield was observed in high-fiber and lower-fat diets but not in low-fiber and higher-fat diets (10). On the contrary, Drago (15) observed improvements in feedlot performance when a higher-fat diet was offered to Nellore bullocks. Thus, taking into consideration that in feedlot nutrition programs higher fiber and lower fat diets are usually fed during the initial and intermediary days of feeding, conversely to the finishing periods, it is necessary to determine which feeding protocol is the most appropriate to start LYSO administration (i.e., adaptation, growing, or finishing diets) and its consequences in dry matter intake (DMI), digestibility, and performance.

Feedlot cattle raised in open dry lots often are exposed to heat events that directly affect animal bioenergetics (16), with the potential to decrease feed and energy intakes, ADG, and efficiency (17, 18). As a result, it is necessary to monitor meteorological variables during the feeding period. To better describe and understand the effects of supplemental feed additives on DMI, especially during heat events, we evaluated and proposed an innovative methodology to monitor the thermal comfort of feedlot cattle.

We hypothesized that fat and fiber digestion, as well as animal performance, would be increased when LYSO was added to the diet of finishing *Bos indicus*-influenced cattle. The objectives of this study were (1) to evaluate three different strategies of LYSO supplementation on feedlot performance, carcass characteristics, nutrient digestion, and the *longissimus* fatty acid profile of *Bos indicus* and *Bos indicus* crosses and (2) to validate an innovative environmental index named InComfort (InCl) as a tool to evaluate DMI during natural heat stress events in the course of conducting open dry-lot feedlot experiments.

## 2. Materials and methods

All procedures and protocols involving the use of animals were approved by the Ethics Committee on animal use of the São Paulo State University “Julio de Mesquita Filho” (UNESP; Protocol number: 016339/19).

Two experiments were simultaneously conducted at the Agro-Pastoril Paschoal Campanelli Research Center in Altair, São Paulo, Brazil (20° 31′26″S 49° 03′32″O; average annual temperature is 22.9°C and annual rainfall is 1,287 mm). The first experiment (Exp. 1) was conducted in a large pen setting with the goal of evaluating performance variables, and the second experiment (Exp. 2) was conducted in electronically monitored pens to access digestive, physiological, and metabolic variables. The experiments were carried out from 1 June to 23 September 2020.

### 2.1. Pre-experimental procedure

From 21 March to 8 May 2020, a total of 1,970 predominantly *Bos indicus* and *Bos indicus* × *Bos taurus* crossbred bullocks weighing 380.9 ± 16.9 kg (mean ± SD) and approximately 24-month-old were selected for the experiment. The cattle originated from 16 different stocker ranches with an average transportation distance of 693 km (minimum 50 km and maximum 910 km) to the Agro-Pastoril Paschoal Campanelli Research Center. At the end of the weighing process, animals were immediately allocated into seven 12-hectare *Cynodon dactylon* grass paddocks equipped with feedlot bunks. A pre-experimental maintenance diet (Table 1) without feed additives was offered *ad libitum*. This diet was formulated to reestablish the ruminal environment and equalize the physiological conditions of all animals before the experiments.

**Table 1:** Diet composition (Experiments 1 and 2).

	**Diets**			
**Item**	**Pre exp^1^**	**Adaptation**	**Growing**	**Finishing**
**Ingredient (%, DM basis)**				
Sugar cane silage	30.1	–	–	–
Sugar cane bagasse	35.8	–	–	–
Corn silage	–	31.5	17.3	9.42
Snaplage	–	19.5	25.9	27.1
Citrus pulp	–	18.7	25.7	31.2
Soybean molasses	14.3	6.80	6.10	6.54
Peanut meal	20.5	10.9	9.46	6.60
Whole cottonseed	–	9.27	10.6	13.1
Protected fat^b^		–	1.38	2.62
Urea	0.91	0.92	0.98	1.00
Trace mineral supplement^a^	2.24	2.27	2.41	2.46
**Diet composition, % of diet**				
DM	52.0	52.0	59.0	65.0
Ash	6.40	3.00	3.00	3.50
CP	11.8	16.0	15.6	14.7
EE	1.20	3.50	5.00	6.50
NDF	52.9	33.5	29.9	27.7
Ca^c^	0.74	1.33	1.70	1.98
P^c^	0.35	0.52	0.43	0.37
NFC	25.7	44.1	46.5	47.7
NEm, Mcal/kg^c^	1.08	1.82	1.89	1.97
NEg, Mcal/kg^c^	0.53	1.19	1.25	1.32

a Calcium 127.9 g/kg, phosphorus 23.0 g/kg, cobalt 25.0 mg/kg, copper 420.0 mg/kg, sodium 40.0 g/kg, sulfur 14.0 g/kg, iodine 25.0 mg/kg, magnesium 15.0 g/kg, manganese 810.0 mg/kg, selenium 15.0 mg/kg, zinc 1,500.0 mg/kg, iron 0 mg/kg, vitamin A 72,000 IU/kg, vitamin D3 14,413 IU/kg, vitamin E 500 IU/kg, monensin 714.0 mg/kg, virginiamycin 714.0 mg/kg.

b Calcium soap of fatty acids, ~86% of fatty acids from soybean and 14% of calcium. Lysoforte^*TM*^ eXtend inclusion was 1.7% in the premix of treated animals, in those of 1 g/1% of EE.,

c Estimated by LRNS.

1 Pre-experimental (maintenance diet); trace mineral premix was the same as NON treatments, without LYSO inclusion.

All animals were ear-tagged, dewormed with an oral drench of 1 ml per 20 kg of body weight (BW) of 10% fenbendazole (Panacur, MSD Saúde Animal, São Paulo, São Paulo, Brazil), and vaccinated against bovine respiratory disease with an intranasal live and attenuated vaccine (1 ml in each nostril; Inforce, Zoetis, São Paulo, São Paulo, Brazil). Additionally, animals were vaccinated against clostridia (5-ml subcutaneous injection; Poli-Star, Valée S/A, Montes Claros, Minas Gerais, Brazil).

On 19 May, animals were submitted to a 16-h feed and water withdrawal. To ensure that all animals were weighed with the same restriction time on a subsequent day to determine initial body weight (IBW), a 15-min staggered-interval schedule for both feed and water restriction was applied. Following weighing, bullocks were blocked according to their respective IBW using a macro lottery spreadsheet with a random number function in Microsoft Excel 2011 (Microsoft Corporation, Redmond, Washington, USA). The 114 animals that presented weight variations two standard deviations above the mean or presented health issues were eliminated from the experiment.

A total of 1,760 animals were selected for Exp. 1, with 890 animals that were predominantly *Bos indicus* Nellore and 860 animals classified *as Bos indicus* × *Bos taurus* crossbred bullocks. To maintain experimental unit homogeneity, both genotypes were equally distributed in each pen (28 *Bos indicus*, predominantly Nellore, and 27 *Bos indicus* × *Bos taurus*).

In parallel to Exp. 1, a total of 96 animals, of which 64 were *Bos indicus* Nellore (NEL, 372.55 ± 20.5 kg) and 32 were *Bos indicus* × *Bos taurus* (½ Nellore × ½ Angus, CEA, 406.42 ± 24.5 kg), with approximately 24-month-old, were similarly processed and selected for Exp. 2. Bullocks were assigned with radio frequency identification ear tags (Allflex, FDX, Joinville, Santa Catarina, Brazil), and, similar to Exp. 1, both genotypes were equally distributed in each of the four electronically monitored pens (16 NEL and 8 CEA).

### 2.2. Design and treatments

A randomized complete blocked design was used for Exp. 1. Bullocks were blocked in four weight groups (8 replications/treatment). For Exp. 2, a completely randomized design with a 4 × 2 factorial arrangement (96 animals, 24 animals per pen, 16 NEL, and 8 CEA) evaluated the same treatments as in Exp. 1 in addition to genotype.

On 1 June, animals from both experiments were switched to the adaptation diet. The following treatments were used: (I) no LYSO supplementation (NON); (II) LYSO supplementation starting at the growing period (GRO), from the 17^th^ day on feed throughout the entire finishing period; (III) LYSO supplementation starting only at the finishing period (FIN), from the 34^th^ day on feed throughout the entire finishing period; (IV) LYSO supplementation during the entire feeding period, starting at adaptation on day 0 to the end of the experimental period (ALL). Prior to the experiments, LYSO was mixed in the mineral premix supplement with a 1,000-L capacity mixer for 15 min, according to the instructions provided by the equipment manufacturer. The LYSO was dosed to provide approximately 1 g of product or 1% of dietary ether extract (DM basis). Mixed samples were sent to a commercial lab to evaluate the mixed quality (CBO Laboratory Analysis, Valinhos, SP, Brazil). This resulted in 3.91, 5.95, and 6.29 g/day of the commercial product (LYSOFORTE^TM^ eXtend, Kemin Industries, Inc., Valinhos, SP, Brazil) in adaptation, growing, and finishing diets, respectively. Monensin (Elanco Animal Health, São Paulo, Brazil) and virginiamycin (Phibro Animal Health, Guarulhos, São Paulo, Brazil) dietary concentrations during the adaptation, growing, and finishing phases were 22.77, 24.20, and 24.70 mg/kg of DM, respectively, for both of these feed additives.

### 2.3. Pens assignment

Animals in Exp. 1 were allocated in open dry lot pens with 0.27 cm of linear bunk space per animal, space availability of 13 m^2^/animal (750 m^2^; 50 m length × 15 m width) with water trough (3.0 m length × 0.8 m height × 0.25 m width). Shade (SH) was provided in all pens (2.4 m^2^/animal). Sheet and cable structures of SH were manufactured with galvalume steel sheet (0.43 mm thickness × 1.08 m wide × 10 m long), 0.15 m of gap distance between sheets, tensioned with a set of eight cables (6.35 mm on top and 3.17 mm under the sheet), and held by carbon steel columns (2.6 mm thickness, 7.62 cm diameter, fixed with a 2-m height concrete base). The sheets were positioned 5 m from the ground in a north-to-south orientation, with an 18° displacement in the northeast-to-southwest direction.

In Exp. 2, animals were allocated to four dry lot pens (375 m^2^, 50 m length × 7.5 m width) with a capacity of 24 steers (15.62 m^2^/animal). These pens were equipped with three electronic feeding system monitors and two individual scales located at the water trough [IS, Model VW1000, Intergado Ltd., Contagem, Minas Gerais, Brazil; (19)]. Troughs were built with a dimension of 3 m length × 0.8 m height × 0.25 m width. Similar to Exp. 1, animals had access to SH, but in this case, 2.7 m^2^/animal was provided to maintain the same steel sheet size according to the respective pen width.

### 2.4. Feeding and health management

For both studies, the experimental feeding program consisted of three diets: adaptation, growing, and finishing. The adaptation diet was fed for 16 days, the growing diet for 17 days, and the finishing diet from 52 to 73 days, according to block IBW (staggered by 7-day intervals from heavier to lighter blocks). The same adaptation and growing programs were offered to the animals in Exp. 2; however, the finishing diet was offered for 69 days.

Diets were formulated to provide nutrients for an ADG of at least 1.5 kg, according to the LRNS [http://www.nutritionmodels.com/lrns.html, accessed May 2020; (20)]. Nutrient levels and diet composition are presented in Table 1. The animals were fed twice daily at 07:30 and 14:00 h, with bunk score calls recorded daily at 06:45 h, following a modification of the method of Pritchard and Bruns (21) adapted for 1–2% of feed refusals. Feed delivery was equally divided between morning and afternoon offers. Individual pen feed refusals were weighed in a staggered manner (5 min/pen) every morning before the first feed delivery with a modified tractor-trailer (Nonino, CAR-Balança, 1,700-kg capacity, Bebedouro, São Paulo, Brazil) equipped with a ±0.10-kg precision electronic scale (Alfa Instrumentos, Samel-2CF, São Paulo). This procedure was adopted to ensure that the bunks always contained feed, considering that the difference between the first and the last pen to be fed was approximately 2.5 h.

Animals were fed using a truck-mounted mixer (Brutale, Model MTB-120CM, 16-m^3^ capacity, São Carlos, São Paulo, Brazil) equipped with an electronic scale (±1 kg precision). The scale was calibrated weekly during the experimental phase. To avoid the confounding effects of feed additives, the mixer was cleaned before every treatment change, four times in the morning and four times in the afternoon. Additionally, it was re-checked for feed residues after the cleaning procedure and flushed with water when necessary. The ingredients were added to the truck-mounted mixer in the following order: corn silage, snaplage, peanut meal, citrus pulp, whole cottonseed, protected fat, soybean molasses, and mineral supplement. Following this, diets were mixed for 4 min before delivery.

One trained person checked animals for signs of disease twice daily. If needed, animals were treated with florfenicol and flunixin meglumine (intramuscular injection, 1 ml per 7.5 kg of BW, Resflor Gold^®^, MSD Saúde Animal, São Paulo, São Paulo, Brazil) for pulmonary issues or with tildipirosin (intramuscular injection, 1 ml per 45 kg of BW, Zuprevo^®^, MSD Saúde Animal, São Paulo, Brazil) when hoof anomalies were detected. If necessary, for both illnesses, a second treatment was given, and the animal was removed from the experiment if recovery was not indicated.

### 2.5. Performance and carcass variables

For Exp. 1, the average DMI was calculated by the difference between offered feed and refusals. Based on that, the DMI of the large pen study (DMI_LP_, kg/animal/day) from each large pen was given as follows:


$$\begin{array}{l} DMI_{j\left(LP\right)}=\left(\frac{OF\left(dm_{f}/100\right)-\;RE\;\left(dm_{r}/100\right)}{NA_{j}}\right) \end{array}$$

where *OF* = offered daily feed and *RE* = refusals were corrected daily to average DM of feed (*dm*_*f*_) and refusals (*dm*_*r*_), and divided by the number of animals in each pen (NAj) (j = 1…,32), respectively, of the i^th^ ordinal day from the experiment (i = 1,…,111).

On the last day of the experimental feeding period, to obtain a final body weight (FBW) measurement, animals were withheld from feed and water for 16 h in a staggered manner as described from the beginning of the experiment. Performance data such as ADG and feed conversion (FC) were calculated based on shrunken IBW and shrunken FBW using the mean DMI of the entire experimental period.

At the end of the experiment, animals were harvested at a commercial packing plant located 330 km from the feedlot. Bullocks were harvested on four separate dates (staggered weekly), according to weight blocks (heavier to lighter). Harvest weight was defined when animals reached 560 kg of shrunken FBW.

Hot carcass weight (HCW) was obtained after evisceration and removal of the kidney, pelvic, and heart fat. Dressing percentage (DP) was calculated as the ratio of HCW to shrunken FBW. As different days of feeding were necessary to reach harvest FBW, the average DP (57.03%) of all animals was also used to estimate adjusted FBW, ADG, FE, and FC. In addition, carcasses were classified by one trained packing plant employee, according to subcutaneous fat deposition (SFD), using five categories (2-, 2= scarce, absence of fat; 2+ and 3- median; 3= and 3+ uniform; 4 excessive; Farol JBS, adapted from Brazil, 2004). After 24 h of chilling, pH was measured in the longissimus muscle (CpH), between the 12th and 13th ribs, using a portable digital pH meter (model HI98163; Hanna Instruments, São Paulo, Brazil) with a puncture electrode (model V-627).

For Exp. 2, IBW was calculated as in Exp. 1. Data were analyzed as repeated measures over time to evaluate the feed additive interactions with meteorological variables. The DMI from each electronically monitored pen was given as follows:


$$\begin{array}{l} DMI_{jk\left(EM\right)}=\;\left(\overset{n}{\underset{i=1}{\sum }}FI_{i\left(EM\right)}\right)\;(dm_{i}/100) \end{array}$$

where *FI*_*i*(*EM*)_ (kg/animal/day) is the daily amount of feed intake in the i^th^ visit in the feed bunk equipped with an electronic feeding system performed by j^th^ animal in the k^th^ ordinal day from feeding period, and *dm*_***i***_ (%) is the percentage of dry matter in the diet.

The live weight of animals without feed and water withdrawal (kg) was registered for every drinking event during all feeding periods. From this data basis, spline functions were adjusted as follows:


$$\begin{array}{l} {w\left(x\right)}_{jk}=\alpha +\beta_{1}x_{jk}+\gamma_{1}z_{1}+\gamma_{2}z_{2}+\gamma_{3}z_{3}+,\ldots ,+\gamma_{19}z_{19} \end{array}$$

where *x* is the kth ordinal day from the feeding period registered in the jth animal, consequently the average daily gain was given as follows:


$$\begin{array}{l} ADG_{jk\left(EM\right)}={w\left(x\right)}_{jk}-{w\left(x\right)}_{jk-1} \end{array}$$

### 2.6. Chemical analyses

Dietary DM adjustments of feed ingredients (corn silage and snaplage) with variable water concentration were conducted twice daily, before feed mixing, throughout the experiment using a Koster Moisture Tester (Koster Crop Tester Inc., Model D, Medina, Ohio, USA). In a similar manner, total mixed diet and feed refusals were collected twice and once daily, respectively. For both variables, treatment composite samples (based on equal amounts of samples per pen) were generated and dried at 105°C [Tecnal, model TE-394/3-MP, Piracicaba, São Paulo, Brazil; method 930.15, (22)] for 12 h to determine DM.

For both experiments, samples of diet, ingredients, and orts were collected weekly, composited, and sampled for chemical analyses. All the samples were dried at 55°C in a forced-air oven for 72 h for DM determination. Dried samples were ground in a Wiley-type mill (1-mm screen, MA-680, Marconi Ltda, Piracicaba, São Paulo, Brazil) and analyzed for ash [method 924.05; (23)], NDF (24), CP (AOAC International, 2012), and EE (method 920.85; AOAC, 1986). The NFC was estimated according to the following equation: NFC (%) = 100% – (% NDF + % CP + % EE + % ash), according to Mertens (25).

Apparent nutrient digestibility was measured in Exp. 2. Indigestible NDF was used as an internal marker, determined by a 288-h *in-situ* incubation procedure (26). Due to the necessary adaptation period of the diet before fecal collection and the fact that during the growing feeding phase, GRO treatment was supplemented with LYSO (similar to ALL) and FIN was not supplemented (similar to NON), it was evaluated NON and ALL treatments, in which was possible to respect animal acclimatation to facilities and human presence, necessary adaptation after dietary change (12 days), and fecal collection period (5 days). Animals from NON and ALL treatments were acclimated to human presence inside pens, during the adaptation feeding period, with the objective to visualize individual animal identification. Starting on the 12^th^ day after animals were transitioned to growing diet, fecal and diet samples from all animals of NON and ALL treatments were collected during 5 consecutive days, hourly staggered, over a 10-h period (from 07:00 a.m. to 05:00 p.m.). Fecal samples were collected directly from the pen floor immediately after defecation (avoiding soil contamination) and individually identified. As a result of a successful acclimation program, it was possible to collect hourly subsamples from 47 animals (23 from NON and 24 from ALL). Composited fecal samples were generated with approximately 10% of the wet weight from each of the hourly subsamples. The same procedure was conducted during the finishing phase, except that it was conducted during the final 5 days of feeding.

The fatty acid profile (FAP) of diets and in the *longissimus* muscle was also evaluated from NON and ALL treatments. Approximately 24-h post-mortem, samples of approximately 2.50 cm were collected from the 12^th^ and 13^th^ ribs of the animals in the second experiment. They were individually identified and vacuum-packed until further laboratory analyses. The FAP was performed according to the methodology described by Folch et al. (27), with the lipid fraction methylated and the methyl esters generated following techniques described by Kramer et al. (28). Qualitative and quantitative measurements of fatty acids were performed *via* gas chromatography (GC-2010 Plus autoinjector – AOC 20i; Shimadzu Scientific Instruments, Kyoto, Japan) using a 100 m × 0.25 mm diameter column of 0.02 μm thickness (Supelco SP-2560; Sigma Aldrich Pty Ltd., Castle Hill, Australia). The initial temperature was kept at 70°C for 4 min, with progressive heating (13°C/min) until the temperature reached 175°C, and this temperature was maintained for 27 min. Thereafter, 4°C/min increases were obtained until the temperature reached 215°C, and this temperature was maintained for 31 min. Hydrogen gas was used as the carrier with a 40 cm^3^/s flux. Fatty acids were identified and quantified, and the peak areas were normalized using software (GC solution) with a standard (non-adecanoic acid; C19:0).

### 2.7. Meteorological data

Solar irradiance (R_S_, W m^−2^; CMP-22, Kipp and Zonen, Delft, Netherlands; spectral range = 0.3–3.6 μm), ultraviolet solar radiation (U_V_, W m^−2^; spectral range = 0.28–0.4 μm), air temperature (T_A_, °C; range = −40 to + 70, accuracy ± 0.1°C, accuracy ± 0.1°C), the black-globe temperature in the sun (T_Gsun_, °C; accuracy ± 0.1°C), relative humidity (R_H_, %; accuracy ± 3%), wind speed (W_S_, m/s; accuracy ± 0.44 m/s), and daily precipitation (P, mm/h) were all continuously recorded every minute using a portable weather station (WS-18 model 110, Nova Lynk, Auburn, CA, USA) placed near the pens. In addition to the meteorological data collected by the weather station, temperature sensors were also placed inside the pens and water troughs and attached to the roof surface of the shade structure for the characterization of the microclimate experienced by the shaded cattle. These local measurements were recorded every 5 min and included air temperature, relative humidity, black-globe temperature, the temperature of the inner surface of the roof, and water temperature.

A set of six black-globe devices was placed in two shaded pens, while three black globes were placed in three unshaded areas, positioned 2 m above the ground surface. Miniature data loggers (i-bottom DS1925L, Maxim Integrated, Sao Jose, US; size = 0.60 × 1.70 cm, height × diameter; accuracy ± 0.5°C) were inserted inside globes for measuring black-globe temperature (T_Gshade1_ and T_Gshade2_, °C) in the shade underneath the roof and exposed to full sun (T_Gsun1_, T_Gsun2_, and T_Gsun3_, °C). Three temperature sensors (i-bottom) were attached to the inner surface of the shade roof structure to obtain the roof surface temperature (T_RS1_, T_RS2_, and T_RS3_, °C). Three temperature sensors (i-bottom) were previously waxed (Sasol wax, GmbH D-20457) and placed inside the water troughs to obtain water temperature (T_W1_, T_W2_, T_W3_, °C). Three temperature-humidity data loggers (HOBO^®^ data logger, model U12-012, Onset Computer Corp., Bourne, MA), of which two were placed inside the shaded pens and one within an unshaded area, were used to obtain air temperature (T_A1_, T_A2_, and T_A3_, °C) and relative humidity (R_H1_, R_H2_, and R_H3_, °C). These temperature-humidity data loggers were shielded against direct solar radiation.

### 2.8. Heat load experienced by feedlot cattle

The principal component analyses (29–33) were used to observe dissimilarities over the days on feed concerning the meteorological conditions (T_A_, H_R_, R_S_, U, W_s_, and T_G_) experienced by feedlot cattle. Principal components were obtained by computing eigenvalues (λ_i_) and the respective eigenvectors $e_{i}^{,}=\left[e_{i1}\;e_{i2}\;e_{i3}\right]$ of the data correlation matrix. The bi-dimensional representation of the multidimensional set was created by using scores for the first (*PCA*_1*j*_ = *e*_11_*T*_*A*_+*e*_12_*H*_*R*_+*e*_13_*R*_*S*_+*e*_14_*U*+*e*_15_*W*_*S*_+*e*_16_*T*_*G*_) and second principal components (*PCA*_2*j*_ = *e*_21_*T*_*A*_+*e*_22_*H*_*R*_+*e*_23_*R*_*S*_+*e*_24_*U*+*e*_25_*W*_*S*_+*e*_26_*T*_*G*_). All principal components were used according to Liu et al. (32) for the development of an environmental index, namely, the InComfort Index (InCI)-Based Membership Function Value Analysis.


$$\begin{array}{l} InCI=\;\overset{n}{\underset{i=1}{\sum }}\left[R\left(\lambda_{i}\right)\;W\left(e_{i}\right)\right] \end{array}$$

where n is the number of principal components and InCI is the weighted membership value calculated with principal components for each day linked with its meteorological conditions, thereby building a ranking with the level of thermal stress. Being *R*(λ_*i*_) given by


$$\begin{array}{l} R\left(\lambda_{i}\right)=\frac{\lambda_{i}-\lambda_{i\left(min\right)}}{\lambda_{i\left(max\right)}-\lambda_{i\left(min\right)}} \end{array}$$

indicating λ_i_ is the value of i^th^ principal component, λ_i(min)_ and λ_i(max)_ are the maximum and minimum values of i^th^ principal component, respectively:


$$\begin{array}{l} W\left(e_{i}\right)=e_{i}/\overset{n}{\underset{i=1}{\sum }}e_{i} \end{array}$$

where *W*(*e*_*i*_) is the weight of the i^th^ principal component among all the principal components selected for evaluating the level of heat stress on animals, and e_i_ is the contribution rate of the i^th^ principal component.

The values of InCI are in an interval from 0 to 1, with the lowest value representing meteorological conditions that are more comfortable for animals. Conversely, the highest InCI values, reflect meteorological conditions that negatively affect the thermal comfort of animals. Based on water intake and respiratory rate, the InCI were divided into four classes, namely, rainy days, when the mean of 0 ≤ InCI ≤ 1, with precipitation rate above 20 mm/day; cloudy days, when the mean of 0 ≤ InCI ≤ 0.4 (A); hot days, when the mean of 0.4 < InCI ≤ 0.6 (N); and very hot days, when the mean of 0.6 < InCI ≤ (Q). Water intake (*WI*_*EMP*_, L/animal/day) from each electronically monitored pen was calculated as follows:


$$\begin{array}{l} WI_{EMP}=\overset{n}{\underset{i=1}{\sum }}\left(\frac{W_{fw}-W_{iw}}{0.997}\right) \end{array}$$

where *W*_*fw*_ and *W*_*iw*_ (kg/day/animal) are the final and initial weights of the animal during water intake in the *n*^*th*^ visit of the animal in the water trough on the *i*^th^ ordinal day of the experiment, and 0.997 is the water constant (kg/L).

### 2.9. Statistical analyses

Before statistical analysis, data were checked for normality, homoscedasticity, and outliers using the PROC UNIVARIATE, evaluating Student and Pearson residuals. For the first study, feedlot performance and carcass characteristics were analyzed using the PROC GLIMMIX procedure of SAS (SAS Inst., Inc., Cary, NC, USA) as a generalized randomized block design. For IBW, FBW, ADG, HCW, DP, CpH, and FAP, animals were considered the experimental unit. Treatment, block, breed, number of days in the receiving pasture, and the treatment × breed interaction were considered fixed effects. Because the treatment × block interaction was not significant (*P* > 0.05), it was removed from the model. Treatment within the pen was considered a random effect. A total of 79 animals were removed from the analysis; 59 because of health problems (pneumonia and/or foot rot; 3.35% of animals) and 20 because of divergence between the research team's notes and the packing plant sequence. For DMI and FC, the pen was considered an experimental unit. For this model, treatment, block, pen, and the treatment × block interaction were considered fixed effects, whereas treatment within a pen was considered a random effect. The Satterthwaite approximation was used to determine denominator degrees of freedom for testing fixed effects in both models. Orthogonal contrasts (NON × ALL + GRO + FIN; ALL × GRO + FIN and GRO × FIN) were performed to compare differences among treatment means, and treatment means were also compared by the PDIFF option of LSMEANS. Differences were declared significant when *P*-value was ≤0.05 and regarded as tendencies when the *P-*value was >0.05 and *P*-value was ≤0.10. Carcass classifications were evaluated by the chi-square test.

For the second study, animals were considered the experimental unit. Raw data from electronic equipment (both bunks and scales) was collected and filtered with algorithms developed by our research group (unpublished data). Performance and physiological data were analyzed as repeated measures over time. Repeated measures were analyzed using mixed model methods based on generalized least squares and a variance component estimation, which were performed by a restricted maximum likelihood (REML) algorithm with a procedure for a linear mixed model (PROC MIXED) of the Statistical Analysis System (34), according to Littell et al. (35). Treatment, breed, treatment × breed, a current day on feed nested within treatment × breed, the current weight of the animal on the respective day of evaluation, and both the duration of meals and the number of bunk visits on the evaluated day were considered fixed effects. Animal nested within treatment × breed was considered the repeated measure subject, and means were compared by the PDIFF option of LSMEANS procedure. In this study, the constructed covariance matrix demonstrated that measures taken close in time did not present the same covariance as measures far apart in time. Based on these results and because Akaike's information criterion (AIC), the AIC corrected (AICC), and the Bayesian information criterion were smaller for the autoregressive moving average (1) covariance structure, verifying the superior fit compared with other covariance structures. As NEL animals differed in IBW in comparison to CEA, it was used as a covariable. For digestibility data, treatment, breed, phase, treatment × phase, and treatment × breed were considered fixed effects, whereas pens nested within a treatment were random. The means were compared using the PDIFF option of LSMEANS. Additionally, for the FA profile, the same model described for digestibility was used, means were compared by the PDIFF option of LSMEANS, and differences were declared significant at a *P*-value of ≤0.05 and regarded as tendencies when the *P*-value was >0.05 and *P*-value was ≤0.10.

## 3. Results

### 3.1. Experiment 1 (large pen trial feedlot performance)

Performance results are presented in Table 2. No differences were detected for IBW (*P* = 0.791). The use of LYSO increased FBW (*P* = 0.022); however, no differences were detected among the LYSO phase-feeding treatments (*P* = 0.191). Regarding ADG, no LYSO supplementation effect was observed (*P* = 0.122). Furthermore, when contrasts were used to compare LYSO-supplemented animals, phase-feeding protocols improved ADG compared with ALL (*P* = 0.007). In addition, the same phase-feeding response pattern was observed when NON was compared with GRO (*P* = 0.089; regarded as a tendency) and with FIN (*P* = 0.012) by comparing the pairwise differences among the means. Feeding LYSO did not alter DMI, regardless of the protocol or feeding period (*P* > 0.05). Animals supplemented with LYSO tended to have a lower FC (*P* = 0.090), but no phase-feeding effect was observed (*P* = 0.4738). Conversely, a tendency was observed (*P* = 0.060) when NON was compared with the pairwise comparisons of the means (GRO).

**Table 2:** Effects of lysolecithin-derived feed additive administration on the performance of *Bos indicus* influenced cattle in Experiment 1.

**Item^b^**	**Treatments^a^**				**SEM**	***P*** **-value^c^**		
	**NON**	**GRO**	**FIN**	**ALL**		**N** × **L**	**A** × **G** + **F**	**G** × **F**
**Performance**								
IBW, kg	400.83	400.82	400.86	401.1	0.561	0.791	0.513	0.933
FBW, kg	564.77	568.38	570.18	567.00	2.206	0.021	0.191	0.357
ADG, kg/d	1.528	1.560	1.574	1.522	0.020	0.121	0.007	0.471
DMI, kg/d	11.23	11.01	11.26	11.23	0.181	0.764	0.710	0.357
DMI, %	2.31	2.27	2.29	2.29	0.040	0.532	0.841	0.803
DMI_adap_, kg/d	10.80	10.60	10.81	10.84	0.225	0.848	0.627	0.500
DMI_grow_, kg/d	11.42	11.40	11.59	11.59	0.159	0.575	0.639	0.404
DMI_fin_, kg/d	11.32	11.05	11.30	11.24	0.217	0.609	0.803	0.427
FC	7.41	7.09	7.19	7.25	0.112	0.090	0.473	0.534
HCW, kg	322.50	323.79	325.03	322.48	1.481	0.241	0.095	0.342
DP, %	57.14	56.99	57.03	56.90	0.210	0.290	0.491	0.817
CpH	5.65	5.66	5.59	5.65	0.021	0.611	0.082	0.581
**Carcass classification (%)**								
2=	5.12	4.27	4.95	4.24				
2+	20.73	23.93	25.94	24.24				
3-	63.17	61.14	56.60	58.82				
3=	10.73	10.66	12.50	12.71				
4	0.24	0.00	0.00	0.00				

a Treatments main effects: N, Control (NON); G, Lysoforte supplementation starting in growing phase (GRO); F, Lysoforte supplementation starting in finishing phase (FIN); A, Lysoforte supplementation in all phases (since adaptation; ALL).

b IBW, initial body weight; FBW, final body weight; ADG, average daily gain; DMI, dry matter intake; DMI_*adap*_, dry matter intake in adaptation feeding period; DMI_*grow*_, dry matter intake in growing feeding period; DMI_*fin*_, dry matter intake in finishing feeding period; FC, feed convertion; HCW, hot carcass weight; DP, dressing percentage; CpH, carcass pH; Carcass classification: 2= reflects excessive lean carcass, 2+, 3-, 3+ desirable fat carcasses and 4 excessive fat carcass.

c N × L = NON × others, A × G + F = ALL × GRO + FIN and G × F = GRO × FIN.

Supplementing LYSO did not affect HCW (*P* = 0.241) under the experimental conditions. Similar to ADG, ALL animals tended to have a lesser HCW (*P* = 0.095) than those on the phase-feeding protocols. Likewise, animals from the FIN treatment tended (*P* = 0.055) to have heavier carcasses compared with NON. Carcass classification was not affected by LYSO supplementation (*P* = 0.689). Moreover, neither the DP (%) nor the CpH (*P* = 0.611) was affected by the LYSO supplementation (*P* = 0.290). The mean DP-adjusted performance variables did not differ (neither tendencies were observed), thus, they were removed from the “Results” and “Discussion” sections.

### 3.2. Experiment 2 (electronically monitored feedlot performance)

**Figure 1:**
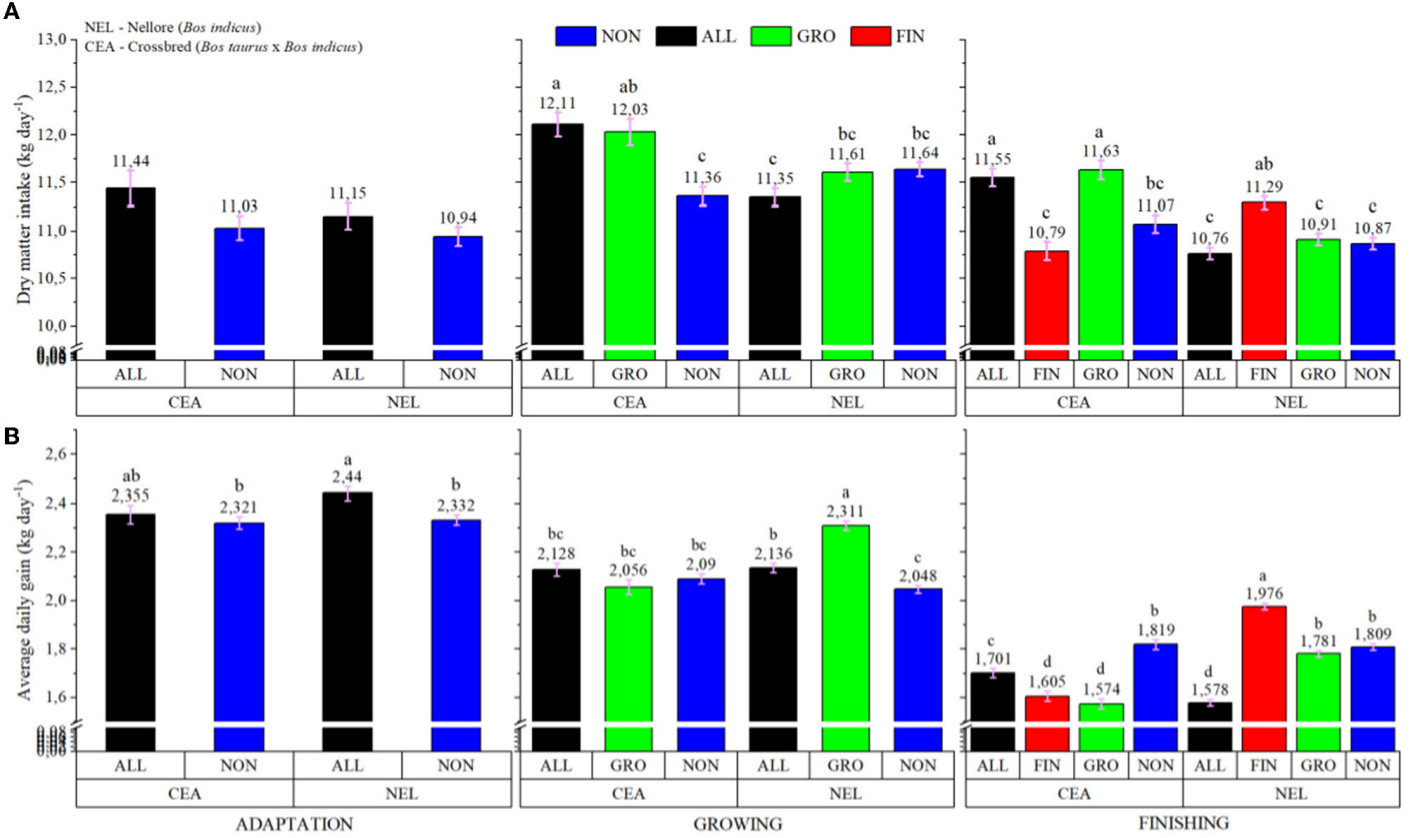
Treatment × Breed × Feeding phase interaction of dry matter intake **(A)** and average daily gain **(B)** of electronically monitored *Bos indicus* influenced bullocks. NON, Control; ALL, Lysoforte supplementation in all phases (since adaptation); GRO, Lysoforte supplementation starting at growing phase; FIN, Lysoforte supplementation starting at finishing phase. CEA = ½ Nellore × ½ Angus, NEL = Nellore. Letters within column with different superscripts differ in LSMEANS at *P* ≤ 0.05.

A treatment × breed interaction was observed, in which CEA animals had a higher IBW (*P* < 0.001; 406.42 vs. 372.55 for CEA and NEL, respectively); however, no differences were detected for FBW (*P* = 0.223; data not shown). Furthermore, a treatment × breed × feeding period interaction was observed for both DMI (*P* = 0.001) and ADG (*P* < 0.001; Figure 1). During the adaptation feeding period, LYSO did not influence DMI regardless of genotype (*P* > 0.05), but during the growing feeding period, CEA bullocks from ALL and GRO had greater DMI intake (*P* < 0.05) than CEA animals from NON and NEL from ALL. During the finishing period, CEA animals from ALL and GRO had greater DMI (*P* < 0.05) than NON and FIN within the same genotype. Moreover, NEL animals from FIN also had greater DMI (*P* < 0.05) than NON, GRO, and ALL cohorts within the same genotype but did not differ from CEA in the ALL and GRO protocols.

The NEL cattle from ALL had greater ADG (*P* < 0.05) than NON animals from both genotypes during the adaptation period. Similarly, during the growing feeding period, NEL from GRO had greater ADG (*P* < 0.05) than NON animals within the same genotype, whereas ALL was intermediate among the NEL cattle. The CEA animals from NON, GRO, and ALL had intermediate ADG. In the finishing feeding period, NEL from the FIN protocol had the greatest ADG (*P* < 0.05), NEL from the GRO and NON protocols (along with CEA from NON) the second greatest, CEA animals from ALL the intermediary, and NEL from ALL and CEA from GRO and FIN had the least ADG.

### 3.3. Experiment 2 (digestibility of nutrients and fatty acid profile of longissimus muscle)

**Figure 2:**
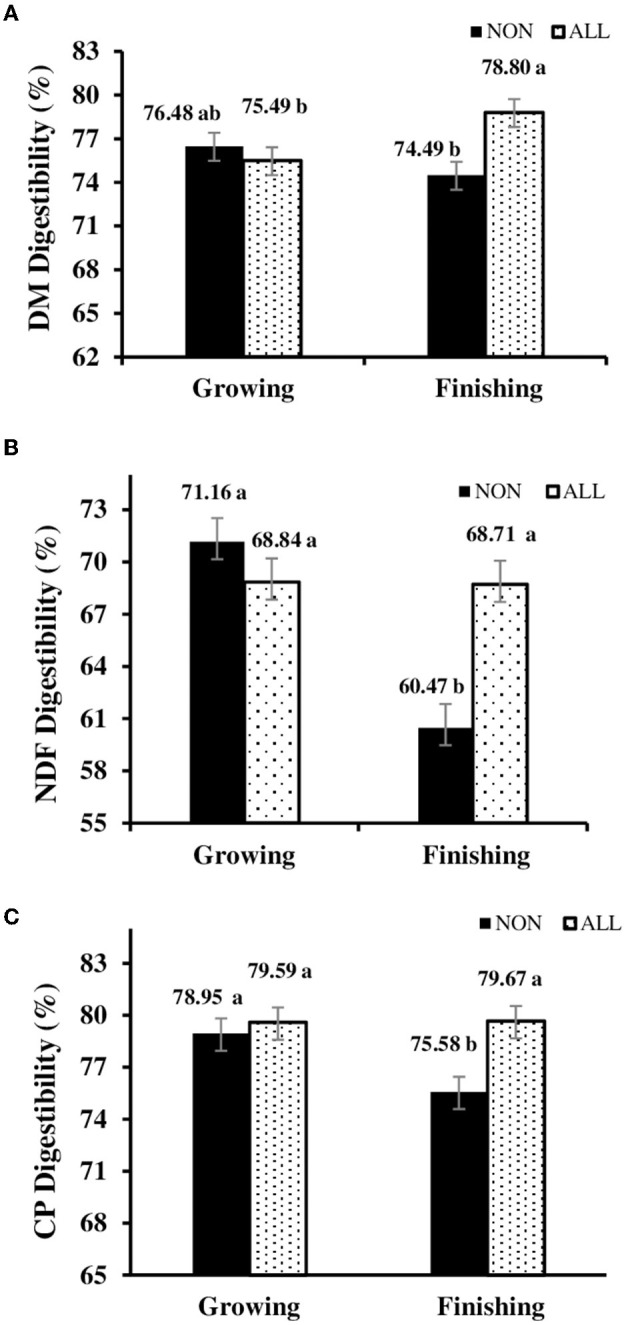
Feeding phase × digestibility interaction [**(A)** dry matter digestibility; **(B)** NDF digestibility; **(C)** crude protein digestibility] of electronically monitored *Bos indicus* influenced bullocks during growing and finishing feeding period. NON, Control; ALL, Lysoforte supplementation in all phases (since adaptation); within column, different superscripts differ in LSMEANS at *P* ≤ 0.05.

No breed effect and breed × treatment interaction was noted for digestibility (*P* > 0.05). However, a treatment-feeding phase interaction was observed (*P* = 0.004; Figure 2). Somewhat surprisingly, no differences were observed (*P* = 0.194) for EE digestibility (81.20 vs. 79.40, NON and ALL, respectively), regardless of the feeding phase. In the finishing feeding period, for ALL protocol, a consistent increase in NDF (*P* < 0.001), CP (*P* < 0.001), and DM (*P* = 0.001) digestibilities was observed.

The FAP of experimental diets is presented in Table 3, and the effects of LYS supplementation during the whole feeding period (ALL) on the *longissimus* FAP are presented in Table 4. Also, no breed effect or breed × treatment interaction was observed for FAP (*P* > 0.05). Supplemental LYSO increased concentrations of C8:0, C17:1, and C18:3n3 (*P* = 0.014, 0.001, and 0.048, respectively), but it decreased those of C20:0 and C24:1 (*P* = 0.004 and 0.003, respectively).

**Table 3:** Fatty acid profile of growing and finishing diets of Experiment 2.

**Fatty acids (g/100g)**	**Growing**		**Finishing**	
	**NON^a^**	**ALL**	**NON^a^**	**ALL**
C10:0 (Capric)	0.018	0.017	0.024	0.036
C12:0 (Lauric)	0.193	0.180	0.247	0.197
C14:0 (Myristic)	0.491	0.513	0.549	0.565
C16:0 (Palmitic)	20.87	21.81	21.62	21.85
C17:0 (Margaric)	0.164	0.082	0.095	0.155
C18:0 (Stearic)	3.557	3.574	4.113	4.257
C20:0 (Arachidic)	0.323	0.303	0.291	0.272
C22:0 (Behenic)	0.243	0.269	0.256	0.247
C24:0 (Lignoceric)	0.245	0.244	0.204	0.182
C16:1 cis 9 (Palmitoleic)	0.376	0.396	0.432	0.475
C18:1:cis 9 (Oleic)	24.59	23.56	23.23	23.19
C20:1 (Eicosenoic)	0.223	0.089	0.480	0.518
C18:2:cis 9 cis 12 (Linoleic)	41.65	42.43	40.79	41.00
C18:3:n3 (Linolenic)	1.814	1.693	1.445	1.683

a NON, Control; ALL, Lysoforte supplementation in all phases (since adaptation).

**Table 4:** Fatty acid profile of *longissimus* muscle of *Bos indicus*-influenced feedlot cattle supplement with lysolecithin-derived feed additive in Experiment 2.

**Fatty acids^a^ (g/100 g)**	**NON**	**ALL**	**SE**	***P*-value**
C8:0	0.004	0.007	0.008	0.014
C10:0	0.052	0.057	0.003	0.311
C12:0	0.059	0.072	0.004	0.075
C14:0	2.534	2.914	0.139	0.063
C15:0	0.243	0.264	0.013	0.285
C15:0 Iso	0.094	0.116	0.008	0.059
C15:0 Anteiso	0.116	0.121	0.011	0.772
C16:0	23.59	24.45	0.464	0.196
C17:0	0.543	0.583	0.028	0.334
C17:1	0.318	0.369	0.009	0.007
C18:0	15.50	14.50	0.623	0.266
C18:1 Trans	34.89	33.46	0.238	0.674
C18:1 Cis 9	32.91	33.65	0.607	0.231
C18:2 Cis 9 Cis 12	85.85	72.92	0.597	0.125
C18:2 Cis 9 trans 11	0.422	0.430	0.027	0.840
C18:2 Trans 10 cis 12	0.012	0.018	0.002	0.124
C18:3 n6	0.019	0.013	0.002	0.091
C18:3 n3	0.302	0.361	0.020	0.047
C20:0	0.070	0.048	0.005	0.004
C20:1	0.156	0.168	0.011	0.431
C21:0	0.046	0.051	0.004	0.440
C22:0	0.066	0.052	0.007	0.152
C23:0	0.033	0.040	0.005	0.319
C24:1	0.145	0.086	0.012	0.002
SFA	43.83	44.07	0.700	0.815
UFA	56.16	55.92	0.700	0.815

a SFA, Saturated fatty acids; UFA, unsaturated fatty acids. NON, Control; ALL, Lysoforte supplementation in all phases (since adaptation).

### 3.4. Experiment 2 (electronically monitored bullocks, breed × treatment × day classification interactions that influence DMI)

**Figure 3:**
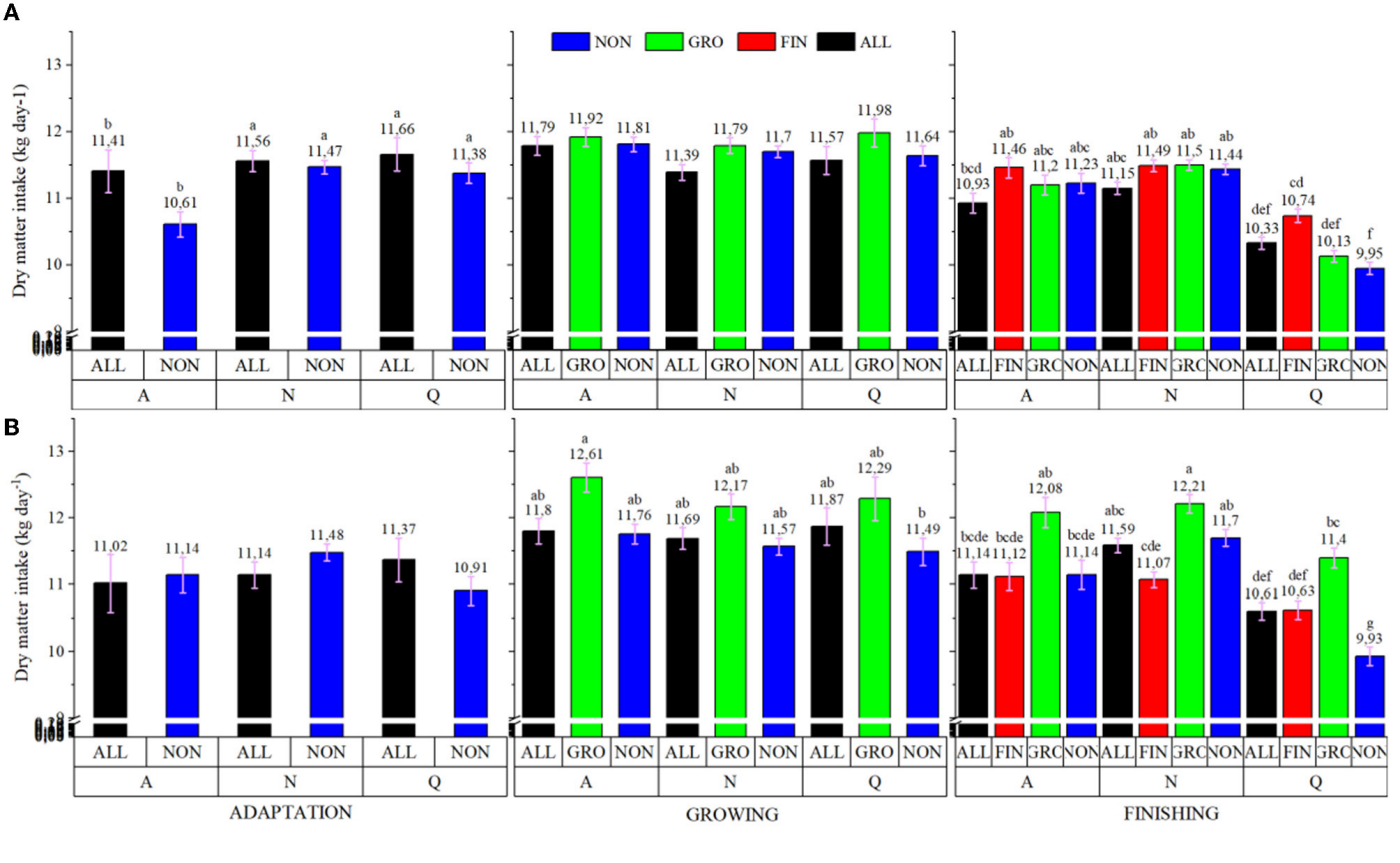
Breed × Treatment × Day classification interaction of dry matter intake of electronically monitored *Bos indicus* (**A** = Nellore; **B** = ½ Angus × ½ Nellore) bullocks during adaption, growing and finishing feeding period. NON, Control; ALL, Lysoforte supplementation in all phases (since adaptation); GRO, Lysoforte supplementation starting at growing phase; FIN, Lysoforte supplementation starting at finishing phase. A, rainy, cloudy mild days; N, normal hot days; and Q, very hot days. Within column, different superscripts differs in LSMEANS at *P* ≤ 0.05.

Meteorological variables are summarized in Table 5. During the experimental period, 37 days were classified as A (mild), 53 days as N (normal/hot), and 10 days as Q (very hot). Based on these classifications, a significant breed × treatment × day classification was observed (*P* < 0.001; Figure 3). There were no differences during the adaptation and growing feeding periods, but the meteorological variables played an important role during the finishing period. Regardless of genotype, DMI drastically decreased when animals experienced Q days during the finishing period. For CEA, bullocks from the GRO protocol had the greatest intake (*P* < 0.05), ALL and FIN were intermediate, and NON was the least. By comparison, NEL from FIN had the greatest DMI (*P* < 0.05), ALL was intermediate, and GRO and NON were the least.

**Table 5:** Meteorological variables of Experiment 2.

**Day classifications^a^**	**Mean**	**Standard deviation**	**Minimum**	**Maximum**
**Class A**				
Air temperature, ^o^C	20.6	2.23	14.7	23.3
Relative humidity, %	65.6	8.00	47.6	86.2
Black globe temperature, ^o^C	23.1	3.08	14.7	27.1
Solar irradiaton, W m^−2^	137.4	66.7	7.32	246.9
Wind speed, m s^−1^	1.39	0.55	0.29	2.73
Wind direction, ^o^	193.4	8.96	103.9	284.5
**Class** ***N***				
Air temperature, ^o^C	22.9	1.77	19.1	27.0
Relative humidity, %	61.0	6.91	45.0	72.8
Black globe temperature, ^o^C	26.8	2.03	23.6	32.2
Solar irradiaton, W m^−2^	195.1	44.0	108.5	33.7
Wind speed, m s^−1^	2.20	0.65	1.21	4.22
Wind direction, ^o^	204.0	44.4	108.4	282.0
**Class Q**				
Air temperature, ^o^C	25.7	1.47	23.0	28.6
Relative humidity, %	54.2	9.52	40.1	68.4
Black globe temperature, ^o^C	30.7	2.42	26.9	36.5
Solar irradiaton, W m^−2^	239.8	51.4	169.9	348.2
Wind speed, m s^−1^	2.87	0.27	1.92	4.54
Wind direction, ^o^	234.2	30.8	168.0	277.5

a A, mild days; N, normal/hot days; and Q, very hot days.

## 4. Discussion

### 4.1. Feedlot performance

The combination of a large-pen study with a smaller-scale, electronically monitored DMI and ADG experiment led to a relevant evaluation of LYSO's mode of action throughout the different feeding phases. In Exp. 1, it was possible to assess the effects of LYSO in a commercial feedlot scenario under a rigorously monitored environment, whereas, in Exp. 2, the mechanism through which the treatments affected performance variables could be better understood within the two most common cattle genotypes fed in the tropics.

Similar to our observations in Exp.1, Zhang et al. (36) reported linear increases in FBW, ADG, and feed efficiency in supplemented Angus cattle without affecting DMI when the dose of a lysophospholipid-derived feed additive was increased from 0.5 to 0.75 g/kg of DM. Moreover, Drago (15) noted improvements in ADG and feed efficiency in LYSO-supplemented Nellore bullocks submitted to a finishing diet with 7.0% EE but not when the diet contained 3.8% EE. Using lambs, Gallo et al. (37) observed 11 and 16% improvements in ADG when the same commercial LYSO product as the one used in this study was tested in diets with 6% of EE in the form of either soybean or sunflower oil, respectively.

The aforementioned studies did not evaluate phase-feeding protocols. Based on the examination showing that animals from NON and ALL protocols had similar performance, the responses observed with LYSO supplementation could be attributed to the GRO and FIN protocols, as supported by the pairwise comparisons of LSMEANS. In addition, GRO and FIN had similar performance, with the former tending to also have better FC and the latter a heavier HCW when both protocols were compared with NON. Therefore, under conditions similar to Exp. 1 (i.e., different genotypes fed in the same pen with greater *Bos indicus* influence), one may assume that starting supplementation of LYSO in growing diets is beneficial in situations when FC is an important driver, whereas the FIN protocol might be more suitable whenever heavier HCW plays an important role. As greater digestibility may be accomplished by the use of LYSO, the additional nutrient uptake might be directed accordingly to the animal's physiological status, resulting in greater ADG and carcass deposition of growing and finishing feedlot bullocks, respectively.

In Exp. 2, the effects of phase-feeding protocols according to cattle genotype were evaluated. First, it is worth noting that CEA cattle started the experiment heavier than NEL cattle. Because CEA cattle have a lighter mature weight than NEL cattle, it is expected that Angus-influenced bullocks start fat deposition at earlier days on feed (38, 39), which also results in a slower rate of gain sooner than NEL cattle.

With regard to CEA animals, DMI was statistically greater for supplemented animals during the growing and finishing feeding phases in the GRO and ALL protocols. This finding suggests that there is a persistent effect of LYSO supplementation and also that intake might be more responsive when stimulated during the initial phases of feeding programs with LYSO for this genotype. For ADG, the lower rate of gain observed for LYSO-supplemented animals during the finishing period suggests that greater fat deposition was expected, changing the composition of gain (40), driven by the higher digestibility of dietary nutrients.

For NEL animals, regardless of DMI for LYSO-supplemented bullocks, during the adaptation and growth phases, statistical differences were only noted during the finishing period. The average daily gain was greater in every phase that LYSO was introduced to the diet, but animals from the ALL protocol presented decreased ADG during the finishing feeding phase. These findings suggest that NEL animals were more physiologically responsive to the dietary digestibility increment, redirecting nutrients for a faster rate of BW gain. Nonetheless, LYSO supplementation beginning in the adaptation feeding period might also anticipate fat deposition and, consequently, change gain composition, in accordance with the ADG results from Exp. 1 and CEA bullocks of Exp. 2.

### 4.2. Nutrients digestibility and fatty acids profile of longissimus muscle

The absence of a breed × treatment effect for both digestibility and FAP suggests that the LYSO mode of action is independent of genotype. The digestibility of EE was not influenced by LYSO supplementation. One plausible explanation is that calcium salts of fatty acids (**CSAF**) from soybeans represented approximately one-third of total dietary EE. High-lipid digestibility in CSAF-supplemented NEL bullocks fed high-concentrate feedlot diets has been previously reported (11, 41). Similarly, Drago (15) reported greater lipid digestibility for CSAF from soybeans in feedlot NEL bullocks. In that study, LYSO did not modify lipid digestion, which suggests that the potential for LYSO to further improve EE digestion was likely decreased when animals are fed rumen-protected highly digestible fat sources. In contrast, Zhang et al. (36) observed a positive linear effect on EE digestion with a lecithin-derived feed additive (up to 8.75 g/animal/day) when rumen-protected fat was added to the diet, but the type and source of fat were not described. The authors suggested that EE digestibility was increased as a result of the effective emulsification of lecithin in reducing the size of fat globules, forming smaller micelles, and, as a consequence, increasing the surface area of lipid droplets for interaction with pancreatic enzymes. The interaction of fat sources (i.e., fatty acid profile and ruminal protection) and inclusion levels with the capabilities of different lysophospholipids deserves further investigation.

Similarly to the NDF digestibility increments observed in Exp. 2, Drago (15) tested the same LYSO product supplemented in this study with three different main sources of fat (CSAF from soybean, CSAF from palm, and degummed soybean oil). The authors reported a 6.81% increment, regardless of the fat source. In addition to increased NDF digestion, Drago (15) also reported improvements in total VFA production and lower ruminal pH for the treatment with the combination of LYSO and soybean oil compared with either CSAF from soybean or palm oil, which indicates that LYSO likely improved ruminal fermentation by facilitating the emulsion and passage of fatty acids out of the rumen, decreasing the potential negative effect that unsaturated fatty acids can have on ruminal microbial function. Higher cellulolytic enzyme activities and enhanced fiber degradation in the rumen have also been noted with the use of synthetic emulsifiers (42).

Crude protein digestibility was positively affected by LYSO supplementation during the final feeding period. Increments in CP digestibility in feedlot cattle supplemented with lysophospholipids have been previously observed (15, 36). The rationale applied in nonruminant animals to explain the effect of lysolecithin-derived emulsifiers with regard to the greater number and size of membrane pores and altered fluidity and transmembrane permeability of nutrients in the intestine may be plausible for ruminants as well (36, 43), but further research is needed. Greater ruminal ammonia was observed in Drago (15) and when lysophospholipid-supplemented cows were compared with cohorts receiving monensin (44). In the later study, lysophospholipid-supplemented animals presented greater purine derivative excretion, which might indicate higher microbial protein supply, N secreted in milk, and reduced urinary N excretion. The authors suggested that dietary N was absorbed in more utilizable forms for protein synthesis in the body, which could partially explain the greater ADG of NEL bullocks fed LYSO as they have higher protein requirements (45). Therefore, the greater DM digestibility and performance observed in this study in response to supplemental LYSO seem to be related to improved CP and NDF digestibility.

The longissimus FAP of feedlot cattle supplemented with either LYSO or other emulsifiers has not previously been described. The most remarkable finding is the greater concentration of alpha-linolenic acid (C18:3n3) and a trend (*P* = 0.091) for linolenic acid (C18:3n6), which are exclusive of dietary origin (46), indicating that LYSO possibly enhanced ruminal escape of these specific fatty acids. In a biohydrogenation-induced milk fat depression trial, Rico et al. (10) concluded that the decrease in milk fat in LYSO-treated cows was not specifically related to biohydrogenation, suggesting that it was associated with substrate emulsification in both the rumen and intestines. Furthermore, the higher concentration of octanoic acid (C8:0), which is derived from branched-chain amino acids [valine, leucine, and isoleucine; (47)], could be partially linked to greater NDF digestibility (48).

### 4.3. Influence of meteorological variables on DMI of LYSO-supplemented cattle

Heat stress has been extensively investigated in countries with a mature feedlot industry like the United States and Australia. In the former, it is estimated that heat stress is annually responsible for US$282 million in losses (49), while in the latter is estimated to cost a total of AU$16 million (50). Nonetheless, long-term financial losses are likely to exceed 5–10 times animal mortality because of decreased DMI and lower performance (51). Deleterious effects of heat stress on intake and digestive parameters in *Bos indicus* animals were only evaluated in calorimetric chambers (52), which may not reliably represent an open dry-lot condition as solar radiance is not measured.

As DMI was affected by heat events in the present research, we suggested that InCl would help to elucidate variation in DMI under research conditions and possibly in commercial feeding operations. Another remarkable aspect of the present methodology is the use of animal physiological variables to evaluate animal comfort, in contrast to regular heat stress induction protocols that artificially alter temperature and humidity.

During the conduct of Exp. 2, 10% of days were classified as Q. A decrease in DMI with heat stress was observed during the finishing feeding period, regardless of genotype, but effects were less in LYSO-supplemented CEA and LYSO-supplemented NEL for the FIN treatment. Thus, one might consider supplementing LYSO during conditions in which DMI might be affected by heat events. To the best of our knowledge, this is the first experiment to look at the effects of emulsifiers on heat stress amelioration in cattle. Digestibility enhancement is plausibly responsible for the improved DMI because the subcutaneous temperature was not altered by the use of LYSO (data not shown). Meneses et al. (52) observed detrimental effects on DMI and DM digestibility and a shift in nutrient digestibility from the rumen to the intestines in heat-stressed NEL heifers. These authors proposed that modifying the site of digestion in NEL animals reflected an adaptive response of the digestive tract to heat stress conditions. As LYSO may improve intestinal digestibility (CP and DM), it could partially explain the lower decrease in DMI. It should also be noted that SH availability may have possibly interfered with the DMI responses of ALL and GRO of NEL during heat events that occurred in the finishing feeding phase. Nonetheless, the mechanisms by which LYSO improved the DMI of CEA animals on very hot days need elucidation, but they might be related to a lower heat increment derived from a more efficient fiber ruminal fermentation.

## 5. Implications

This study's findings suggest that LYSO enhances feedlot performance when administered during the growing and/or finishing feeding phases of *Bos indicus*-influenced cattle. The emulsification increased CP, NDF, and DM digestion of the finishing diet and also increased ruminal escape of dietary fatty acids. Finally, the development of the InCl index helped to identify DMI alterations during heat events, and LYSO supplementation ameliorated decreases in DMI caused by heat stress.

## Data availability statement

The original contributions presented in the study are included in the article/supplementary material, further inquiries can be directed to the corresponding author.

## Ethics statement

The animal study was reviewed and approved by Committee on Animal Use of the São Paulo State University Julio de Mesquita Filho.

## Author contributions

RP: conceptualization, conduction, data curation, writing, and editing. JO: writing, data curation, and editing. GM: data curation and editing. MC: writing. LG: conceptualization and data curation. JC and RC: conceptualization, diet formulation, and conduction. PC and MN: conduction. VA and AM: conduction and data curation. VC: conceptualization, intellectual input, and conduction. LSC and LBC: analysis conduction (digestibility). DL: fatty acids profile analysis. MG: editing and revising. All authors contributed to the article and approved the submitted version.
